# A new software tool for computer assisted *in vivo* high-content analysis of transplanted fluorescent cells in intact zebrafish larvae

**DOI:** 10.1242/bio.059530

**Published:** 2022-12-13

**Authors:** Jan-Lukas Førde, Ingeborg Nerbø Reiten, Kari Espolin Fladmark, Astrid Olsnes Kittang, Lars Herfindal

**Affiliations:** ^1^ Centre for Pharmacy, Department of Clinical Science, University of Bergen, 5021 Bergen, Norway; ^2^ Department of Internal Medicine, Haukeland University Hospital, 5021 Bergen, Norway; ^3^ Department of Biological Sciences, University of Bergen, 5006 Bergen, Norway; ^4^ Division for Haematology, Department of Medicine, Haukeland University Hospital, 5021 Bergen, Norway; ^5^ Department of Clinical Science, University of Bergen, 5021 Bergen, Norway

**Keywords:** Acute myeloid leukaemia, Myelodysplastic syndrome, Confocal imaging, Cell segmentation, Automatic image processing, Zebrafish

## Abstract

Acute myeloid leukemia and myelodysplastic syndromes are cancers of the bone marrow with poor prognosis in frail and older patients. To investigate cancer pathophysiology and therapies, confocal imaging of fluorescent cancer cells and their response to treatments in zebrafish larvae yields valuable information. While zebrafish larvae are well suited for confocal imaging, the lack of efficient processing of large datasets remains a severe bottleneck. To alleviate this problem, we present a software tool that segments cells from confocal images and track characteristics such as volume, location in the larva and fluorescent intensity on a single-cell basis. Using this software tool, we were able to characterise the responses of the cancer cell lines Molm-13 and MDS-L to established treatments. By utilizing the computer-assisted processing of confocal images as presented here, more information can be obtained while being less time-consuming and reducing the demand of manual data handling, when compared to a manual approach, thereby accelerating the pursuit of novel anti-cancer treatments. The presented software tool is available as an ImageJ java-plugin at https://zenodo.org/10.5281/zenodo.7383160 and the source code at https://github.com/Jfo004/ConfocalCellSegmentation.

## Teaser

**Summary:** We present a software tool for automatic cell segmentation of fluorescent cancer cells in zebrafish larvae to determine characteristics of the cancer cell population on a single-cell basis

## INTRODUCTION

Cancer remains a major cause of death in both industrialised and developed countries, despite intensive research. This can partly be explained by the complexity and heterogeneity of the disease, but also due to lack of good models for drug screening. This is particularly valid for the myeloid malignancies acute myeloid leukaemia (AML) and myelodysplastic syndromes (MDS). While alternative treatments such as hematopoietic stem cell transplantation (HSCT) and immunotherapies like CAR-t-cell therapy exist, most patients rely on chemotherapy at some time during the treatment. A challenge in the treatment of MDS and AML as well as many other cancers is that elderly or patients with poor general condition are unsuited for curative treatments like HSCT and have to resort to chemotherapy (Liu, 2021; Saygin and Carraway, 2021). Moreover, these patients also have low tolerance for intensive chemotherapy. For AML, the median survival is 5 to 10 months in elderly and frail patients, and for high-risk MDS patients, the only remaining treatment is hypomethylating agents like azacitidine (Aza), which gives an increased median survival of 10 months compared to supportive care (Döhner et al., 2015; Fenaux et al., 2009). While some new treatments are in clinical trials or have been approved in the last decade, such as Enasidenib for AML and MDS patients with IDH_2_ mutation, a demand for novel treatments persist (Cazzola, 2020). Taken into consideration the heterogeneity of AML and MDS, and cancer in general, there is a need for more tailored therapies. However, to facilitate rapid drug development, there is a dire need for relevant models that allow for testing of for instance a novel drug candidate on a large number of various cancer cells representing the different sub-classes of the disease. At present, this is not feasible using rodent models due to price and ethical considerations, and *in vitro* models are not able to fully recreate the complex microenvironment that the cancer cells reside in.

Zebrafish have become an intriguing tool for the development of anti-cancer therapies. The species shares around 70% of the human genes and has orthologues for approximately 80% of proteins linked to human diseases (Molina et al., 2021). Compared to mammalian disease models like mice, zebrafish possess several benefits such as high fecundity rate, low maintenance cost and ease of genetic manipulation. Zebrafish are also translucent during the early stages of development or even throughout adulthood in the genetically modified zebrafish line Casper, which enables the use of optical microscopy techniques on living subjects (White et al., 2008). Especially well-suited for drug screening are zebrafish during the embryonal and larval period, due to their aforementioned optical transparency, small size, and absence of an adaptive immune system until 4 to 6 weeks post-fertilization, while still having important and relevant anatomical structures and physiological processes (Molina et al., 2021; Sullivan et al., 2017).

In cancer research, observing the response of cancer cells to treatments, as well as interactions between the cancer and host, is highly valuable. By injecting fluorescently labelled cancer cells in zebrafish embryo and larvae, these interactions can be observed using confocal microscopy. While zebrafish embryos and larvae are well suited for microscopy, processing of the acquired data remains a significant bottleneck (Mikut et al., 2013). Automated processing methods can enable larger scale high-content screenings using confocal microscopy where the manual data processing and analysis limits the amount of information that can be mined. Some solutions of this problem are already devised (Carreira et al., 2021; Yamamoto et al., 2019); however, these solutions are optimised to work on 2D microscope images. This approach struggles to identify individual cells if these are overlapping in the image plane, hence it identifies fluorescent areas instead of individual cells. This causes some shortfalls, for instance inaccurate determination of cancer cell locations and colocalization with other fluorescent elements of interests due to the lack of one spatial coordinate. Additionally, agglomerations of fluorescent cell fragments or debris may be mistaken for cells since only the total fluorescent area is considered instead of the size of individual objects.

The aim of the present work was to develop a software that can improve the field of automatic image analysis for zebrafish cancer models compared to previously published tools (Carreira et al., 2021; Yamamoto et al., 2019). Our approach processes 3D confocal images and segments individual cells, which enables high-content image analysis for zebrafish larvae models of myeloid malignancies, as well as other cancers. The software tool was evaluated by investigating the proliferation of AML and MDS cells in zebrafish embryo and larvae, and their response to the drugs daunorubicin (DNR) and Aza determined. We illustrate the use of single cell detection to extract additional information from confocal images, such as cell volume distributions and *in vivo* cell density maps. To evaluate the potential cardiotoxic effects of the administered treatments, we utilised a simple algorithm to automatically detect the larva heart rates from 10-s microscope videos.

## RESULTS AND DISCUSSION

### Cell segmentation

A large amount of data are contained in images obtained by confocal microscopy. However, this information can quickly be lost during data processing due to the feature extraction from 3D confocal images being very time consuming and highly cumbersome. To alleviate this problem, we developed a software tool that automatically segments cells and further extracts relevant information from the acquired images. Here, we injected zebrafish larvae with fluorescently labelled leukaemia cells at 2 days post-fertilization (dpf) and imaged daily using confocal microscopy until 5 dpf. While Kimmel et al. defined the transition between the embryonic and larval stages to occur at after the protruding-mouth stage at 72 h post-fertilization, for convenience and to avoid confusion, we use the term zebrafish larva for all stages from the day of injection at 2 dpf (Kimmel et al., 1995).

**Fig. 1.**
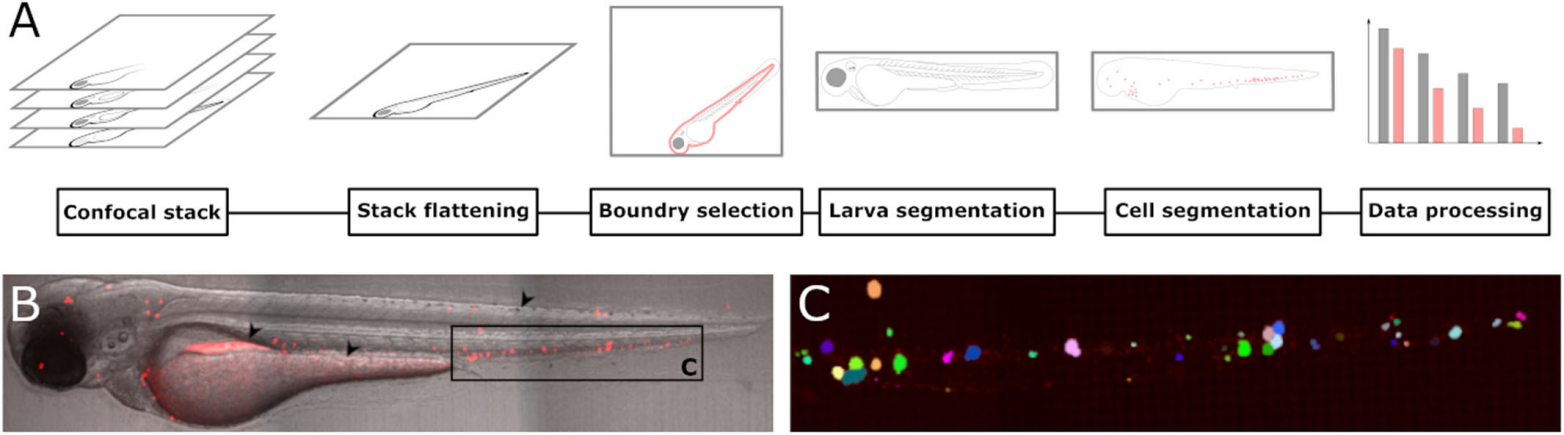
**Processing of confocal images derived from zebrafish larvae intravenously injected with fluorescent cells.** The workflow for segmenting cells from confocal images is illustrated in A. Acquired confocal images are first flattened to a 2D representation to enable easy visual analysis. Following flattening, the larval boundaries are selected by the user to determine the location and orientation of the larva. Using this information, the larvae are segmented and realigned to a standardised orientation. Background levels are determined by the user and all objects located within the larva are segmented using a watershed algorithm. Next, the data can be exported for further analysis. An example of the cell segmentation is given in B and C. A zebrafish larva was injected with 4 nL of a 10 10^6^ cells·ml^−1^ CellTracker™ Deep Red-stained cancer cell suspension into the posterior cardinal vein at 2 dpf. A 2D representation of a confocal image acquired the day after cell injection is shown in B. Common sources of autofluorescence that need to be masked prior to segmentation are the gut, yolk sack and iridophores as indicated by black arrows. Cell segmentation of the tail region (indicated by the black rectangle in B) is displayed in C. To illustrate the segmentation, each segmented object detected is represented by a unique colour.

An overview of the segmentation process is given in Fig. 1A. The process starts by flattening the confocal stack to a 2D representation using a user selected projection method for the fluorescent channel, such as a max-projection, and stack sharpening for the brightfield channel. This is followed by the selection of larval boundaries for alignment as well as background removal. Following larval segmentation, the user can adjust background levels as well as masking sources of autofluorescence to enable the segmentation of fluorescent objects within the larva. While the 2D representation of the confocal stack is used during background adjustment, for cell segmentation the entire 3D stack is used. Alternatives to manual background removal can be achieved using techniques such as the iterative threshold approach used by Carreira et al. (2021). However, such approach did not yield satisfactory results in our data due to excessive removal of weakly fluorescent cancer cells or inclusion of regions with high autofluorescence. Alternatives to intensity-threshold based methods like fluorescence lifetime imaging could be applied for background removal. Where such a technique is not available, computational methods can be utilised. Automatic removal of background fluorescence could be performed using machine learning or by including additional factors, such as location within the image, comparison of multiple image channels and/or edge detection within the segmentation algorithm, however, development of these methods is beyond the scope of the work presented here.

Fig. 1B shows a typical confocal image following stack flattening and larva segmentation. While the area outside the larva is visible in this image, during cell segmentation, only fluorescent objects within the larvae are included. The larva depicted was injected with Molm-13 cells stained with the fluorescent marker CellTracker™ Deep Red Dye at 2 dpf and imaged using confocal microscopy the following day. Common areas of auto fluorescence include the gut, yolk sack and iridophores as marked by black arrows in Fig. 1B. These areas can however readily be masked during the cell segmentation process. Fluorescent objects above the user-set background level are segmented using a watershed algorithm. This results in the segmentation of single objects even if multiple objects are in contact, as long as a border between the objects is detectable in the fluorescent channel. To illustrate this, Fig. 1C shows the segmentation of single objects within the framed area marked with ‘C’ in Fig. 1B. Each object is given a unique colour to illustrate successful segmentation.

The cell segmentation performed by our tool also enables collection of additional data for individual cells such as fluorescent intensity in all acquired channels, volume, and precise location within the zebrafish larva. If the images are flattened before analysis, which is the case for many other analysis tools, information is lost, and quantitative and qualitative analysis of single cells is limited. For instance, information of fluorescent intensity could be used to monitor proliferation of the cancer cells, since the fluorophore concentration in cells is halved for every division. This enables the distinction between treatments that kill cancer cells and those that lead to senescence, as the latter would retain a higher fluorescent intensity compared to dividing cells. An illustration of plots comparing cell volumes to the average fluorescence of cells is given in Fig. S1 for Molm-13 cells in untreated and DNR-treated zebrafish larvae. These plots demonstrate that cell fluorescence decrease over time, correlating with cell division, and further that cell size decreases in DNR-treated larvae. The latter suggests the presence of apoptotic cells. Additionally, since the boundaries and positions of cells are known, cut-outs containing single cells can be extracted from the original confocal image and further analysed in a manner similar to imaging flow cytometry.

### Identification of viable cancer cells based on volume distributions

When imaging fluorescently labelled cells in zebrafish larvae, not every fluorescent object may represent a cell. Apoptotic bodies or cell fragments can still retain the fluorescent stain and be misidentified as viable cells even after being phagocytosed by macrophages. A method for separating these objects from viable cells can be achieved by using volume information to distinguish larger cells from smaller fragments.

**Fig. 2.**
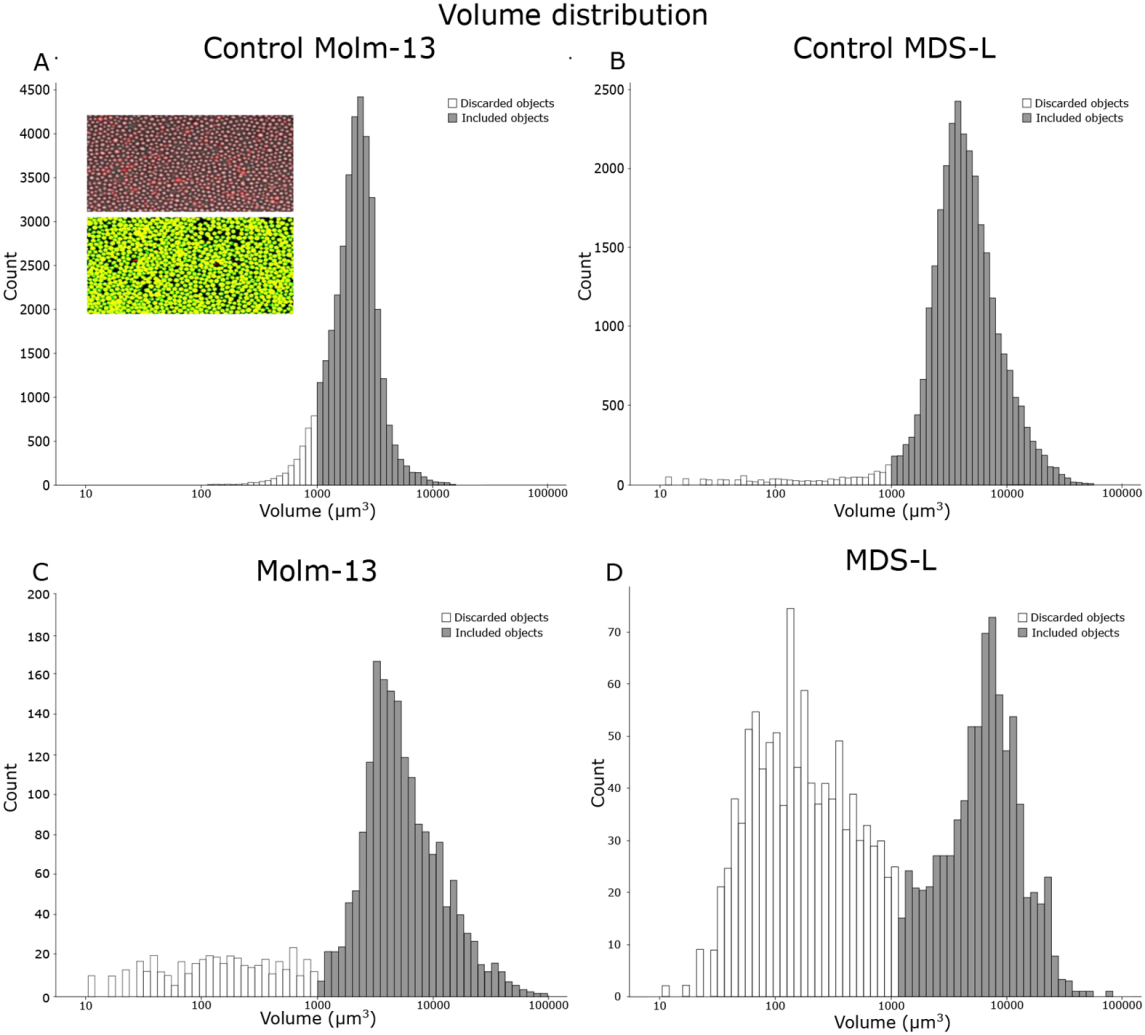
***In vitro* and *in vivo* measurement of cancer cell volume distributions based on confocal images.** Cultured Molm-13 AML and MDS-L cells were stained with CellTracker™ Deep Red and imaged by confocal microscopy (A and B, respectively). An illustration of the segmentation process is shown as inset in A, with the composite fluorescence and brightfield image shown at the top and the resulting segmentation below (composite image of red fluorescence and segmented overlay in green). The plots illustrate the volume distribution with objects below the volume threshold of 1000 µm^3^ shown in white and above in grey. For volume distributions of cancer cells in zebrafish larvae, 4 nL of 10 10^6^ cells·ml^−1^ CellTracker™ Deep Red-stained cancer cell suspensions were injected into the posterior cardinal vein of 18 zebrafish larvae at 2 dpf. Following cell injection, the larvae were imaged by confocal microscopy, and the images processed as the control samples in A and B. Volume distributions for the cell lines Molm-13 and MDS-L in zebrafish larvae are given in C and D, respectively. Using the volume distribution from A and B, a lower volume cut-off for viable cells was determined. The cell populations above and below this threshold are shown in grey and white, respectively. The plots are combined numbers from 3 images in A and B, and 9 larvae for each group in C and D. An illustration of the inter-larvae variation is given in Fig. S3.

To determine the lower threshold volume for viable cells, a volume distribution was created using the segmented objects of all injected larvae at the day of cell injection. As a comparison to cells in zebrafish larvae, cells stained with CellTracker™ Deep Red were kept in medium and imaged using confocal microscopy. The resulting volume distributions are shown in Fig. 2. Based on these volume distribution plots, the volume thresholds for viable cells were determined to be above ≈1000 μm^3^ for both Molm-13 and MDS-L. This volume responds to an equivalent spherical diameter of above 12 μm. The measured size can however be impacted by scattering in the z-plane when acquiring confocal images. This will cause objects to appear stretched along the z-axis and can impact accurate measurement of volumes. However, all measured objects are affected in a similar manner, and their relative differences are still readily detectable. Compared to Molm-13, the size distribution of MDS-L exhibited two populations at the day of injection (Fig. 2D). This population, consisting of objects with smaller volume as shown in white, could represent fragments from apoptotic cells. Such fragments will rapidly be phagocytosed by macrophages, and the small fluorescent objects seen in Fig. S2 could therefore be both free apoptotic bodies, as well engulfed material inside macrophages. It is apparent that Mom-13 cells tolerate the handling prior to injection better than the MDS-L judged by the relatively large amount of sub-cellular fluorescent particles present in the MDS-L-injected larvae (Fig. 2D).

### Distribution of injected Molm-13 and MDS-L cell lines in zebrafish larvae

**Fig. 3.**
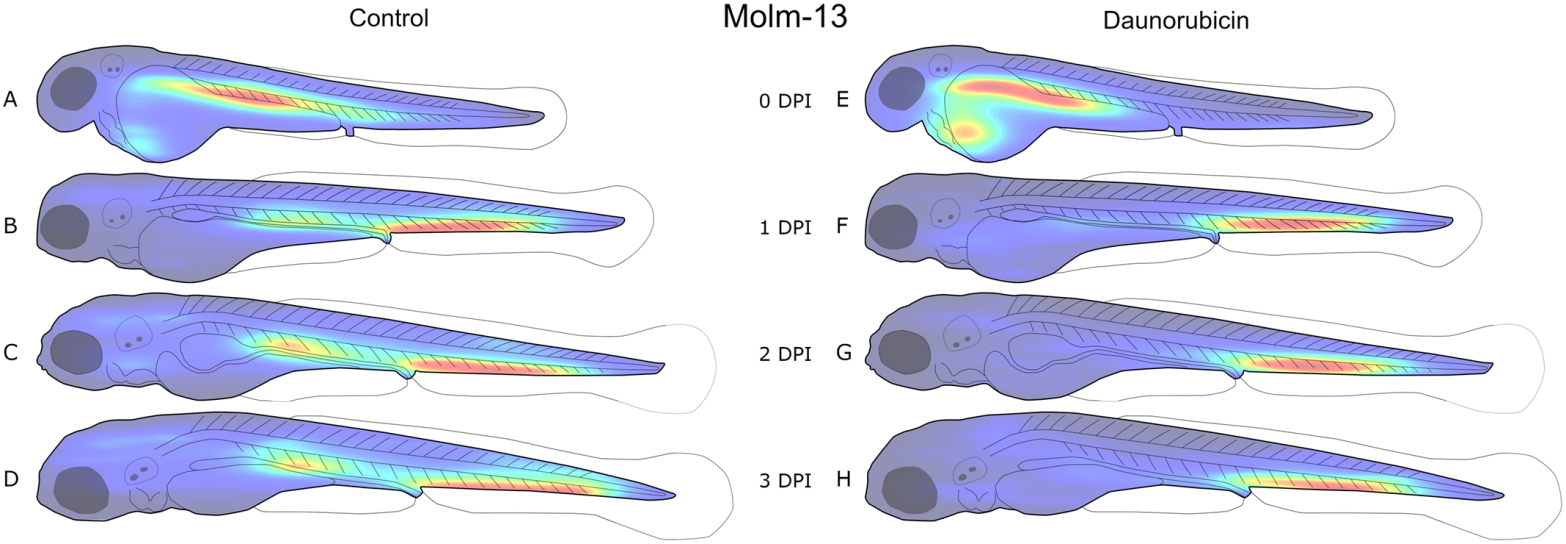
**Distribution of Molm-13 cells in zebrafish larvae after intravenous injection with daunorubicin.** 4 nL of a 10 10^6^ cells·ml^−1^ CellTracker™ Deep Red-stained Molm-13 cell suspension was injected into 18 zebrafish larvae at 2 dpf, and either left untreated (A-D, *n*=9) or treated with a 4 nL injection of 1 mM daunorubicin (E-H, *n*=9). Each larva was imaged daily using confocal microscopy, and the images analysed using our ImageJ plugin as described in the Materials and Methods section. The location of each cell above the volume threshold determined in Fig. 2C was used to create a combined distribution map for each group on each dpi. The density map was generated using MATLAB and visualises the areas of highest (red) and lowest (blue) cell density. Each density map is normalised to its own highest and lowest values; thus, it only visualises distribution, not total tumour burden. An illustration of the variation between replicates is given in Fig. S4.

**Fig. 4.**
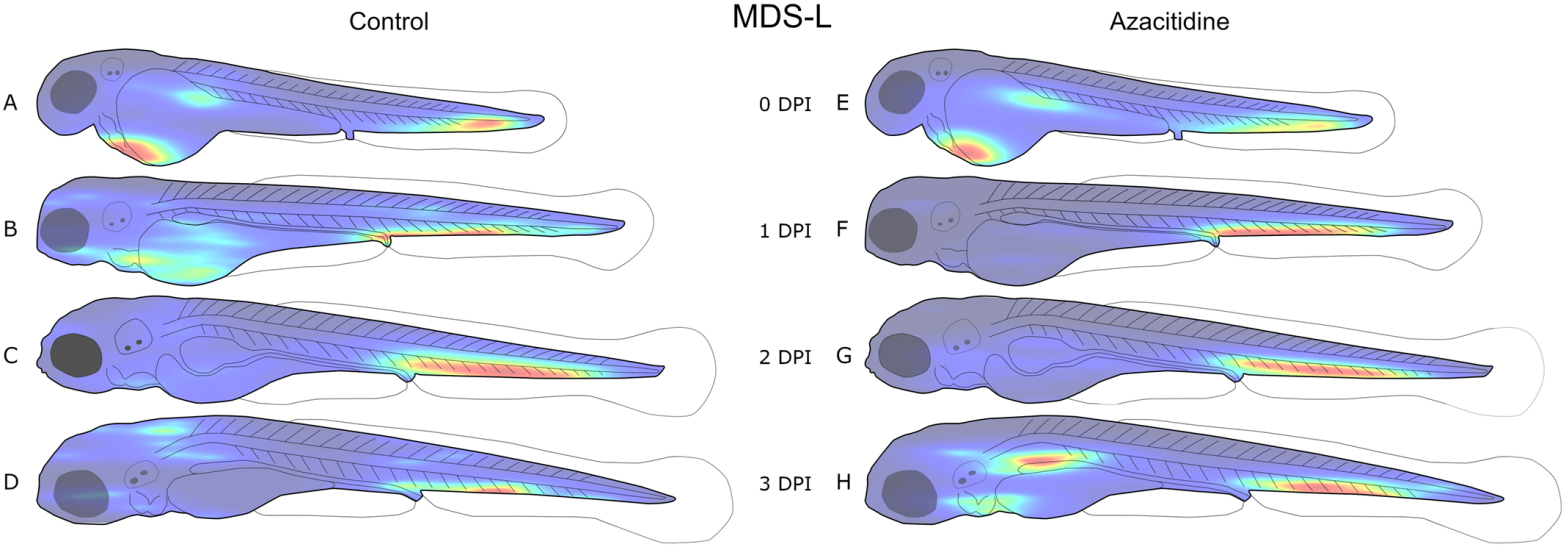
**Distribution of MDS-L cells in zebrafish larvae after intravenous injection with azacitidine.** 4 nL of a 10 10^6^ cells·ml^−1^ CellTracker™ Deep-Red-stained MDS-L cell suspension was injected into 18 zebrafish larvae at 2 dpf, and either left untreated (A-D, *n*=9) or treated with daily 4 nL injections of 1 mM azacitidine (E-H, *n*=9). Each larva was imaged daily using confocal microscopy, and the images analysed using our ImageJ plugin as described in the Materials and Methods section. The location of each cell above the volume threshold determined in Fig. 2D was used to create a combined distribution map for each group on each dpi. The density map was generated using MATLAB and visualises the areas of highest (red) and lowest (blue) cell density. Each density map is normalised to its own highest and lowest values; thus, it only visualises distribution, not total tumour burden. An illustration of the variation between replicates is given in Fig. S4C and D.

The precise position of each detected object with a volume above the predetermined threshold of 1000 μm^3^ was recorded during cell segmentation. Using this data, density maps of injected Molm-13 or MDS-L cells in zebrafish larvae were constructed as seen in Figs 3 and 4, respectively, with Fig. S4 illustrating variations in the distribution between the larvae. It is important to note that each of the density maps in Figs 3 and 4 are normalised to the highest value within that map, and not to the other treatments. This makes each distribution clearer; however, changes in cell counts between density maps are not visualised

At the day of injection, a high cell density can also be seen around the injection site in the posterior cardinal vein in Molm-13 (Fig. 3A,E), and in the heart region for MDS-L (Fig. 4A,E). From 1 day post injection (dpi) until the end of the experiments (3 dpi), both cell lines accumulated in the ventral tail region around the caudal vein. This region houses the caudal haematopoietic tissue (CHT) from 2 to 5 dpf (Gore et al., 2018). A possible explanation for this accumulation could be a selective exit from the bloodstream by the cancer cells at a haematopoietic niche due to cytokines which also attract human leukaemia cells, whose natural environment is the bone marrow.

The Molm-13 cell distributions obtained from larvae treated with DNR (Fig. 3E-H) show a lower cell density around the posterior cardinal vein compared to the untreated transplanted control, while the population in the CHT remains strong. The reduction of cancer cells around the posterior cardinal vein can be a result of the injection site of DNR being in the same area; however, the high injection velocity of the drug, as well as a functioning circulatory system likely result in a rapid and even distribution of the drug in the blood. Thus, a more likely explanation is that the AML cells are more protected from DNR when residing in the haematopoietic niche in the CHT.

In larvae injected with MDS-L, the regions outside the CHT were found to have a higher cell density in control larvae without injection compared to larvae treated with Aza (Fig. 4A-D and E-H, respectively). A notable outlier can be observed in Fig. 4H, where a high cell density can be found around the posterior cardinal vein in Aza-treated larvae when compared to control larvae (Fig. 4D). The MDS-L cells are therapy resistant, and the new population around the posterior cardinal vein could be the formation of a new colony outside the CHT, presumably in the kidney, which takes over as the main haematopoietic tissue at around 4-5 dpf (Paik and Zon, 2010).

### Quantification of tumour burden in zebrafish larvae

**Fig. 5.**
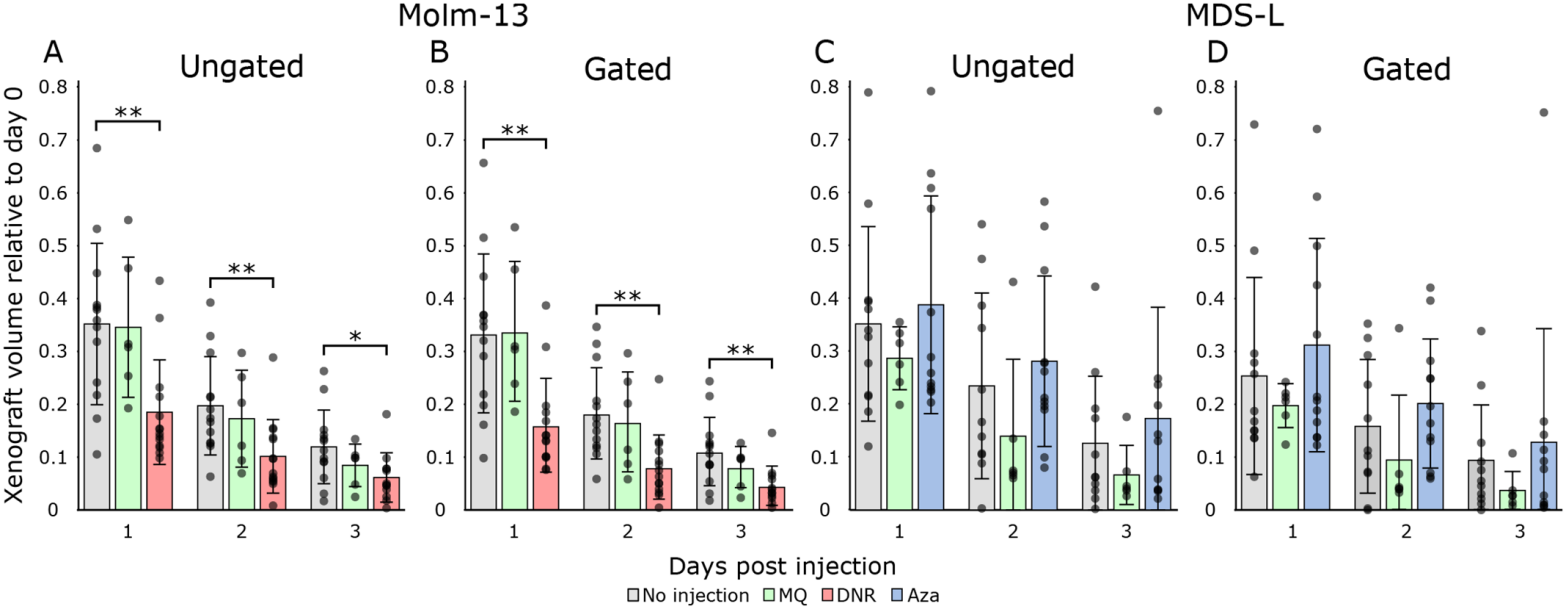
**Tumour burden of Molm-13 and MDS-L in zebrafish larvae with or without anti-cancer treatment.** Molm-13 and MDS-L cells were stained with CellTracker™ Deep-Red and 4 nL of a 10 10^6^ cells·ml^−1^ suspension engrafted into zebrafish larvae 2 dpf by injection into the posterior cardinal vein. Following engraftment, the larvae were imaged daily using spinning disk confocal microscopy. Treatment of Molm-13 cell-xenografted larvae consisted of a single 4 nL 1 mM daunorubicin injection at the day of the transplant, whereas MDS-L engrafted larvae were given daily injections of 4 nL 1 mM azacitidine. Control samples consisted of transplanted larvae without injection as well as larvae injected with 4 nL Milli-Q^®^ water at the day of transplantation for Molm-13 and daily for MDS-L. Imaging and treatment were continued until the larvae reached 5 dpf. Using our software tool, fluorescent cells were counted and segmented based on the confocal images. The total volume of all segmented objects in larvae engrafted with Molm-13 and MDS-L are shown in A and C, respectively. Using the volume threshold of 1000 µm^3^ determined from the images in Fig. 2C and D, the filtered total cell volume was determined (B and D). *n*=15 except for MQ-injected larvae, where *n*=6. Significance between injected larvae and non-injected controls were found using two-tailed Welch's *t*-test. Not annotated: *P*>0.05, *P≤0.05, **P≤0.01.

As well as determining the positions of segmented cells, the software tool also measures cell count and volumes. Using this information, the temporal development of tumour burden was compared in larvae transplanted with Molm-13 or MDS-L. The larvae were then given anti-cancer treatment, blank injections, or no treatment (Fig. 5). We observed a decline in tumour burden in untreated larvae throughout the 3-day observation period. This can be due to the stress the cells are subjected to from handling before and during the injection, as well as the temperature change from 37°C in the incubator to 31°C in the zebrafish. The decrease in cell numbers was highest during the first 24 h, and less dramatic at 2 and 3 dpi for Molm-13 (Fig. 5A,B) with mitotic activity being evident as illustrated in Fig. S2. This indicates that the cancer cells have adapted to the new microenvironment. Such an initial decline in engrafted cells is also seen in mammalian cancer models such as mice, where patient derived xenografts in immunosuppressed mice have an initial latency period followed by proliferation (Siolas and Hannon, 2013).

The combined volumes of up to 16 Molm-13 injected larvae given different treatment, without and with volume gating are shown in Fig. 5A and B, respectively. As seen in Fig. 5B, larvae treated with DNR (red) displayed a significantly lower tumour burden compared to untreated larvae (grey) throughout the observation period. Injection with Milli-Q^®^ water (green) did not lead to a significant decrease in tumour burden. Since the fluorescence is also visible in apoptotic cells, it is important to exclude small cell fragments to be able to quantify the amount of living cells. An impact on the volume distribution of Molm-13 cells due to the DNR treatment can also be seen as shown along the y-axis of Fig. S1. The combined cell volumes of MDS-L cells without and with volume gating are shown in Fig. 5C and D, respectively. In contrast to Molm-13 cells treated with DNR, no significant difference in total tumour burden can be seen in MDS-L cells treated with Aza. This can be attributed to the slow mechanism of action for hypomethylating drugs as observed in *in vitro* studies, but also that the MDS-L cell line is therapy resistant, and not likely to respond to this drug.

### Monitoring cardiotoxic effect from the anti-cancer drugs Daunorubicin and Azacitidine

The use of zebrafish larva as a model for cardiotoxic drugs has been demonstrated in several studies (Maciag et al., 2022; Zhu et al., 2014). Usually, the cardiotoxic effects are monitored by manually counting the heart rate. In addition to being time-consuming, this can also lead to biased results if not performed blindly. However, by using a software to count the heart rate of zebrafish larvae, both these issues are eliminated. The macro used for these measurements uses intensity fluctuations in the acquired microscope videos due to the larva's heartbeat. A detailed description of this is given in the Materials and Methods section.

**Fig. 6.**
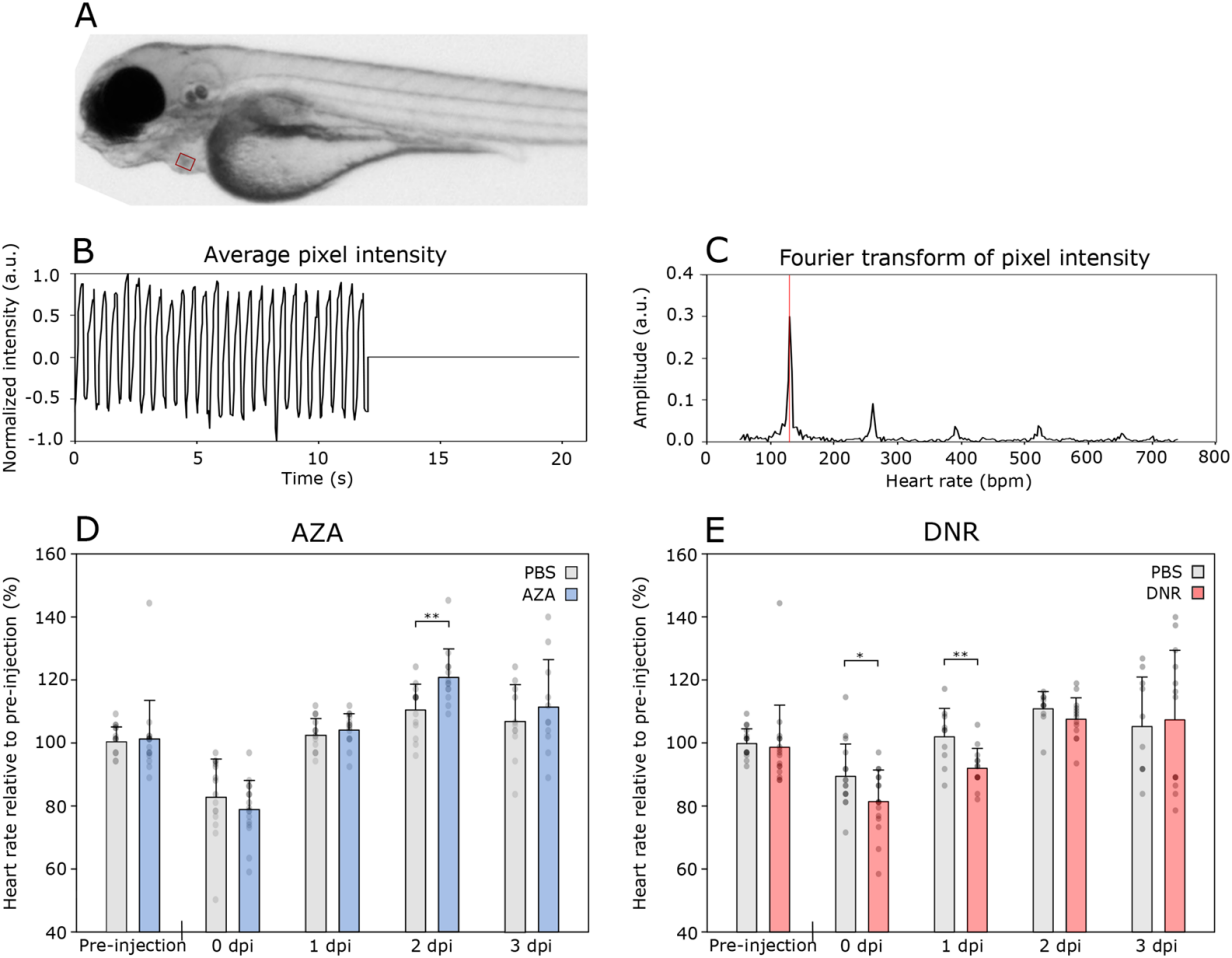
**Aza and DNR effects on zebrafish heart rate.** The zebrafish larvae heart rate in the cardiotoxicity assays was found by filming the zebrafish for 12 s and using a self-written ImageJ macro to calculate the heart rate from the obtained video as described in the Materials and Methods section. (A) Still image from a film analysing zebrafish larvae heart rate. The red rectangle marking the region of interest (ROI) for pixel intensity measurement. (B) Average pixel intensity in the ROI. (C) Fourier transform of pixel intensity from the ROI to calculate beats per minute (BPM). (D) Heart rate of zebrafish larvae injected with 4 nL PBS (*n*=11) or Aza (1 mM, *n*=11). (E) Heart rate of zebrafish larvae injected with 4 nL PBS (*n*=10) or DNR (1 mM, *n*=13). For the Aza-test, the zebrafish larvae were injected daily after Pre-injection, 1 dpi and 2 dpi measurements. For the DNR-test, the zebrafish larvae were injected once, after the Pre-injection observation. The heart rate was related to the average heart rate of the control group pre-injection. Significance found using two-tailed Welch's *t*-test. Not annotated: *P*>0.05, *P≤0.05, **P≤0.01.

Previous studies have shown that anthracyclines reduce heart rate in zebrafish larvae (Han et al., 2015), and we wanted to test whether we could observe this effect using our macro for calculating heart rate. A reduction in heart rate for DNR-treated larvae occurred only at 1 dpi, but not at 2 and 3 dpi (Fig. 6D). This could be because of elimination of DNR by metabolism or excretion. Also, zebrafish are known to be able to regenerate tissue, and heart regeneration has been observed in adult fish (Poss et al., 2002).

In the fish treated with Aza, there was an increased heart rate at 2 dpi (Fig. 6E). The effect could be a stress reaction of multiple injections of the cytostatic drug. In contrast to DNR, the effect of Aza on the heart of zebrafish is little studied, but one report showed reduced survival, malformations and cardiac effects such as pericardial oedema and reduced ventricular volume at 3 dpf when injected with the drug at the 1 to 4 cell stage (Yang et al., 2019). In humans, cases of pericardial effusion and pericarditis have been reported as a result of Aza treatment (Goo et al., 2019; Newman et al., 2016). In our tests, pericardial effusion as an oedema surrounding the heart was observed in 2 out of 12 of the Aza recipients at 3 dpi, but none of the recipients of PBS injections (Fig. S5). This could indicate that the heart of the zebrafish larvae responds to Aza in a similar manner as the human heart.

In this study we performed intravenous injections of DNR and Aza. Administration of drugs through the embryo water is a more frequently applied method, and while administration through water could give a much more predictable and stable drug concentration, it is not always known to which extent the different drugs are absorbed through the skin of the zebrafish. Moreover, Aza is highly unstable in aqueous solutions and for our purpose not a viable route of administration.

### Conclusion

To be able to fully exploit the multitude of different data available from a high-content model like zebrafish, it is imperative with an analytical tool which efficiently process raw data into quantitative or qualitative measurement. Precise information on essential parameters like total tumour burden, location of cancer cells, tumour development over time, and size and intensity of fluorescent objects, is needed to for instance evaluate the efficacy of a therapy, or to understand disease progression. Whereas previous analytical software programs are efficient with respect to automation and time, loss of data during processing occurs for instance in the process of image flattening. This can result in reduced ability to analyse single-cell parameters like size, intensity, or precise location. The software tool presented in this paper performs automatic segmentation of fluorescent objects without losing information like size, volume, fluorescent intensity, or spatial distribution in the 3D space. While the software was designed for the analyses of leukaemia cells, it can readily be used to analyse other fluorescent objects, for instance zebrafish cells expressing fluorescent proteins or other xenografted cells. The software can also be adapted to analyse any confocal image stack containing fluorescent objects. Since there are only a few steps which require user interaction, namely setting the outline of the zebrafish larvae, and removing background fluorescence, the risk for subjectivity or user-generated errors in data analyses is also reduced. In the future, we envision that also definition of the larva outline and removal of background fluorescence can be automated as well. Then, the need for user interaction in data analyses is eliminated, which will further increase the efficiency and quality of data acquisition from confocal images.

## MATERIALS AND METHODS

### Materials

Microinjection pipettes for cell transplantation (VESbv-11-0-0-55) and microinjection pipettes for drug injection (VICbl-4-0-0-55), were from BioMedical Instruments (Zöllnitz, Germany). Daunorubicin (DNR, Cerubidine) was from Sanofi-Aventis (Paris, France). Azacitidine (Aza, A2385), RPMI-1640 Medium (R5886), Foetal bovine serum (FBS, F7524), L-glutamine (G7513), Penicillin-Streptomycin (P0781) were from Sigma-Aldrich (St. Louis, MO, USA). Ethyl 3-aminobenzoate methanesulfonate (Tricaine E10521) was from Merck Life Sciences (Darmstadt, Germany). Recombinant Human IL-3 Protein (IL-3, 203-IL-010) was from BioTechne (Minneapolis, MN, USA) and CellTracker™ Deep Red Dye (C34565) was from Thermo Fisher Scientific (Waltham, MA, USA).

### Methods

#### Zebrafish maintenance

The transparent zebrafish (*Danio rerio*) line Casper was used (White et al., 2008). Fertilised zebrafish eggs were obtained from the zebrafish facility at the University of Bergen. This facility is run according to the European Convention for the Protection of Vertebrate Animals used for Experimental and Other Scientific Purposes. The zebrafish eggs, embryos and larvae were kept in Petri dishes with E3 medium (5 mM NaCl, 0.17 mM KCl and 0.33 mM MgSO_4_) added methyl blue at 28.5°C. Petri dishes were cleaned daily by removing debris or dead embryos/larvae. After injection of cancer cells, the zebrafish larvae were kept at 31°C as a compromise between the 28.5°C preferred by the larvae and 37°C the injected cells. All zebrafish larvae were euthanised at 5 dpf. For euthanasia, the zebrafish larvae were first kept on ice for at least 20 min before being frozen at −20°C over-night.

#### Cell line maintenance

The AML cell line Molm-13 (Matsuo et al., 1997). and MDS cell line MDS-L (Rhyasen et al., 2014) were cultured at 37°C in a 5% CO_2_ atmosphere in RPMI medium. The medium was enriched with 10% FBS, 20 mM L-glutamine, 100 IU ml^−1^ penicillin and 0.1 mg ml^−1^ streptomycin. For the MDS-L cells, the medium was additionally enriched with IL-3 to a concentration of 25 ng ml^−1^. During maintenance, both cell lines were kept at a concentration between 100.000 and 1.000.000 cells·ml^−1^.

#### *In vivo* cell experiments

### Cell staining

The cells were stained using CellTracker™ Deep-Red Dye following the manufacturer's protocol. In brief, cells were centrifuged at 90 RCF for 5 min, the medium removed, and the cells resuspended in fresh serum free RPMI medium with 20 μM CellTracker™ staining solution. The cells were incubated with the CellTracker™ staining solution for 30 min at 37°C before being centrifuged at 90 RCF for 5 min and resuspended in fresh medium to a concentration of approximately 10 million cells·ml^−1^ for injection into zebrafish larvae.

### Transplantation of human leukaemia cells in zebrafish larvae

**Fig. 7.**
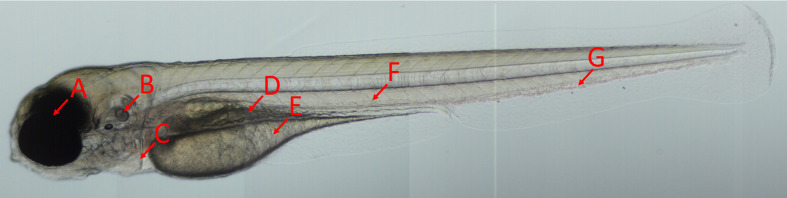
**Overview of the zebrafish larva.** A zebrafish larva imaged at 4 dpf using bright field microscopy. The arrows indicate the eyes (A), otolith (B), heart (C), gut (D) and yolk sac (E). Intravenous injections were performed in the posterior cardinal vein (F) down-stream from the caudal vein (G).

Zebrafish larvae at 2 dpf in the long-pec stage according to the developmental stages proposed by Kimmel et.al., were anesthetised using a 0.7 mM tricaine solution and mechanically dechorionated under a microscope using forceps (Kimmel et al., 1995). A microinjection pipette with an inner diameter of 11 µm was filled with CellTracker™ Deep Red-stained cell suspension and mounted onto a Narishige MMN-5 with MMO-220A (Narashige, Tokyo, Japan) micromanipulator system and connected to an Eppendorf FemtoJet 4x microinjector (Eppendorf, Hamburg, Germany). The injection pressure and time were adjusted to achieve an injection volume of 4 nL. The anesthetised zebrafish larvae were placed on a 2% agarose bed and injected into the posterior cardinal vein as illustrated in Fig. 7. After injection, the zebrafish were kept under anaesthesia and transferred to 18 well chamber slides (µ-Slides, Ibidi, Gräfelfing, Germany). Imaging was performed on an Andor Dragonfly 505 confocal system (Andor Technology, Belfast, Northern Ireland) equipped with an inverted Nikon Ti-E microscope using a Nikon CFI Plan Apochromat lambda 10x objective (Nikon, Tokyo, Japan). The brightfield channel was used to visualise the larva, while a 700/38 nm band-pass filter with a 637 nm excitation laser was used to track the injected cancer cells.

After confocal imaging, half of the larvae were additionally injected into the posterior cardinal vein with an anti-cancer drug, using the same protocol as for cell injections apart from using an injection pipette with an inner diameter of 4 µm. DNR injections were only performed once on the same day as cell injections, while Aza was injected daily after each confocal imaging due to the molecule's short half-life in aqueous solutions. The dosage of DNR was chosen to be below that which could severely affect heart function, but still show significant activity in *in vitro* cell assays. For Aza-treatment of MDS-L transplanted larvae, we only could use the maximal tolerable dose, since the MDS-L cells are therapy-resistant. Transplanted larvae injected with the same volume of MQ were also imaged to find if injection itself affected cell growth.

The following days, the zebrafish larvae were imaged daily using confocal microscopy. Prior to reaching 120 h post-fertilization (5 dpf), all larvae were euthanised. The acquired confocal images were analysed using the self-written software tool.

#### Software tool for image processing

A plugin for the image processing software ImageJ (Version 1.53c; Schneider et al., 2012) was written in Java using JDK 1.8. The plugin was written using the integrated development environment Apache NetBeans IDE 13. A graphical overview of the software tool's workflow is given in Fig. 1A.

The software starts by flattening each confocal stack to a 2D representation. Importing the confocal image files is enabled using the Bio-Formats library (Linkert et al., 2010). The brightfield channels are flattened by utilizing code from the Stack-Focuser plugin originally written by Michael Umorin, but slightly modified to suit our needs (https://imagej.nih.gov/ij/plugins/stack-focuser.html). Fluorescent channels are flattened using a max projection. Following flattening, the borders of the larvae are manually selected by the user. Using the selected outline, the angle of the larva is determined by the selection boundary's Feret angle, while position of the yolk sac is determined using a distance map by identifying the furthest internal point from the boundary. Using the position and orientation of each larva, a montage can automatically be created for each larva from the day of injection to the day of euthanasia.

For cell segmentation, the level of background fluorescence is determined manually by the user, as well as masking of any sources of high autofluorescence, such as iridophores, the gastrointestinal tract and yolk sac. Cell segmentation is performed using a watershed algorithm found in the mcib3d library, with the code slightly modified to increase segmentation speed in our application (Ollion et al., 2013). To find starting points for the watershed algorithm, the fluorescent stack is first pre-processed using a 3D-median filter with radius 3 for x and y, and 2 for z. This is followed by a local maximum filter with a cut-off value of the predetermined background fluorescence. Using the obtained seed image as a starting point, a watershed is performed on the fluorescent stack. This process is repeated for each larva for each daily acquisition, and the detected objects stored.

Following cell segmentation, a size distribution of each detected object is created. From this distribution, a size range for viable cells is determined. Using this size range as a filter, the cell count and total cell volume in each larva is exported for further analysis.

From the location-data obtained from the segmentation process, a heatmap is constructed to visualise cell distributions. X- and Y-coordinates of segmented cells for all larva within the same group and day are combined and a heatmap generated using MATLAB (R2021 Update 2) with a modified version of the Data Density Plot plugin supplied by Malcolm McLean (https://www.mathworks.com/matlabcentral/fileexchange/31726-data-density-plot). All heatmaps are normalised to facilitate the visualization of the cell distribution.

#### Cardiotoxic assay

2 dpf zebrafish larvae were intravenously injected with the drugs. To ensure that the effect of temperature and tricaine was similar in all larvae, the zebrafish larvae were exposed to 0.7 mM tricaine and left in room temperature for at least 30 min prior to recording heart rate. The zebrafish larvae then were injected with either of the following into the PCV: 4 nL DNR (1 mM), Aza (1 mM) or PBS as control. Due to the rapid decomposition of Aza, the injection of this drug and PBS for the representative control group was repeated at three and four dpf. DNR and its PBS control group was only injected at 2 dpf.

To determine heart rate, each larva was filmed for 12 s using a Leica M205 stereo microscope fitted with Leica DFC3000 G camera and the Leica Application Suite X software. At 2 dpf, the zebrafish larvae were filmed both before (Pre-injection) and directly after (0 dpi) the injection. This was to observe whether there were immediate toxic effects of the drugs. For simplicity, the time points are related to the first injection for all groups.

The heart rate was found using a self-written macro for ImageJ. To determine the heart rate, a region around the heart is selected (Fig. 6A). The macro measures the average intensity in each frame of the video and normalises the values to a range between -1 and 1. To comply with the requirements of the fast Fourier transform (FFT) algorithm utilised by ImageJ, artificial measurements with the value zero are added to the measurement list until the total number of measurements equals a power of two (Fig. 6B). After performing the FFT, the heart rate is determined to be the fundamental frequency of the plot (Fig. 6C).

### Statistical analyses

The data in all bar charts is presented as average with standard deviation. Statistical significance between groups was determined using a two-tailed Welch's *t*-test performed using RStudio for Windows, version 2022.02.2 Build 485 (RStudio, PCB, Boston, MA, USA).

## Supplementary Material

10.1242/biolopen.059530_sup1 Supplementary information
