# Supplementary material for: A new software tool for computer assisted in vivo high-content analysis of transplanted fluorescent cells in intact zebrafish larvae
Source: Biol Open. 2022 Dec 13;11(12):bio059530. doi: 10.1242/bio.059530 (PMC9770244; doi:10.1242/bio.059530)
Supplement: Supplementary information [file biolopen-11-059530-s1.pdf]

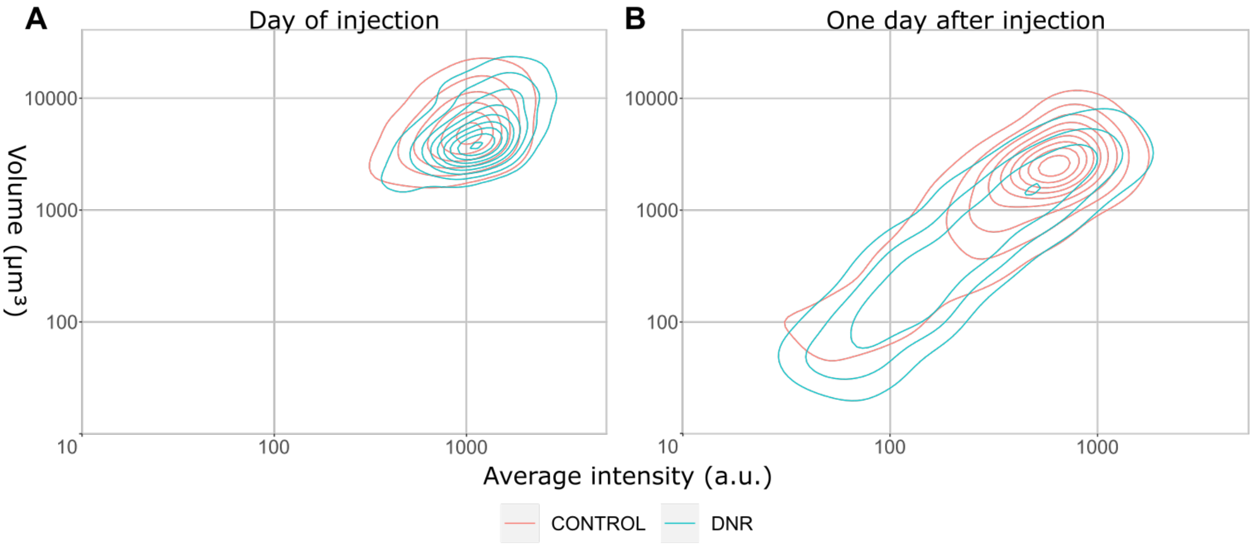

Fig. S1. Density plot of fluorescent intensity vs volume of molm-13 cells transplanted in zebrafish. Zebrafish embryos were injected with approximately 4 nL of a $10 \cdot 10^6$ cells·ml^{-1} CellTracker™ Deep Red stained Molm-13 cell suspension at two days post fertilization, and either left untreated (red) or were treated with a 4 nL injection of 1mM daunorubicin (blue). The larvae were imaged using confocal microscopy at the day of injection and the following day. Using our software tool, the volumes and average intensities of the transplanted cells were measured. The figure above illustrates a 2D density plot of the average intensity and cell volume at the day of transplantation (A) and the following day (B). The data are combined from 15 larvae in each group.

Biology Open • Supplementary information

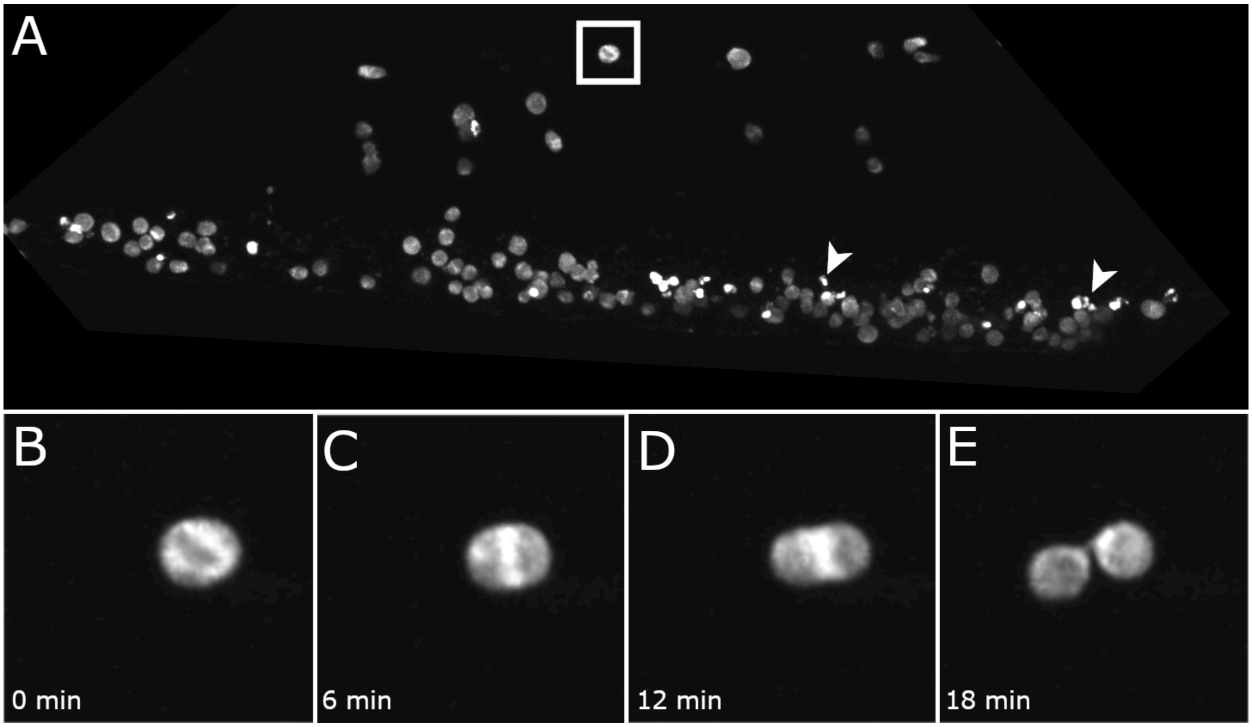

Fig. S2. Cell division of transplanted Molm-13 cells in zebrafish. Zebrafish larvae at two days post fertilization were injected with 4 nL of a 10^6 cells·ml^{-1} CellTracker™ Deep Red stained Molm-13 suspension. The day after injection, the caudal veins of the larvae were imaged every six minutes for 15 hours using confocal microscopy to detect mitosis. An overview of the caudal vein of a larva is given in a max-projection of the fluorescent channel in A. The arrows indicate clusters of apoptotic bodies, possibly engulfed by macrophages. Cell division is marked with a white box is illustrated in B to E. which are successive frames of the time lapse, with 6 minutes between each frame.

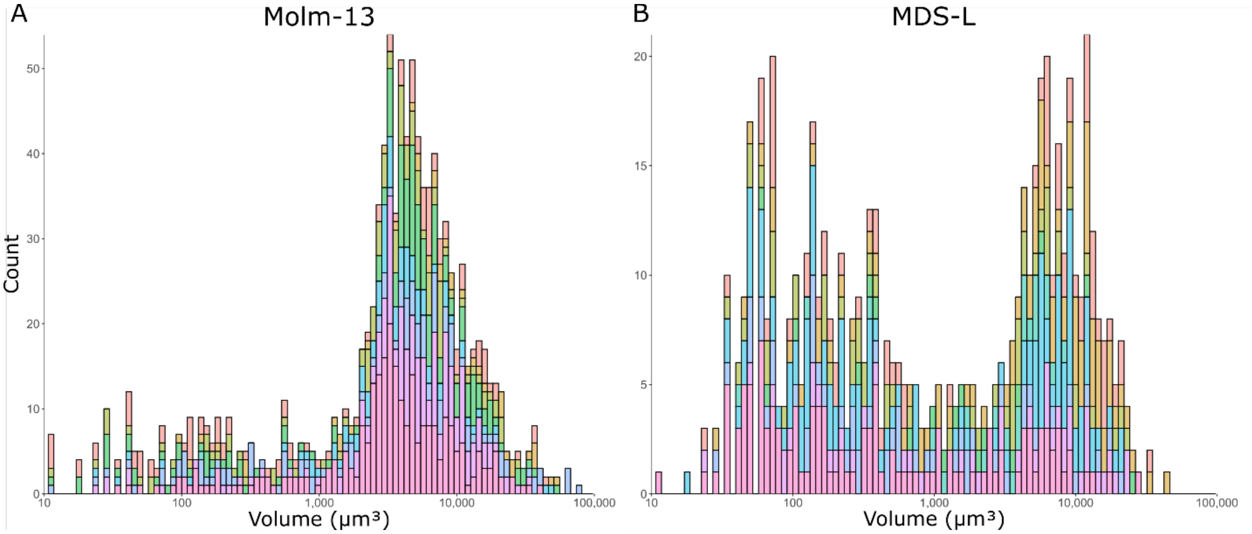

Fig. S3. Inter-larva variations in volume-distribution in transplanted leukaemia cells. Four nL of a $10 \cdot 10^6$ cells·ml^{-1} cell suspension containing CellTracker™ Deep Red stained Molm-13 or MDS-L cells were injected into the posterior cardinal vein of zebrafish larvae at two days post fertilization. Following cell transplantation, the zebrafish larvae were imaged using confocal microscopy. Using our software tool, the cell volumes determined, and a volume distribution created. The variations in the volume distributions are illustrated above as a stacked histogram with each larva represented in a different colour. Larvae transplanted with Molm-13 are given in A, MDS-L transplanted in B. The data shows the number of fluorescent objects at different sizes in the nine larvae of the untreated group for each cell line.

Biology Open • Supplementary information

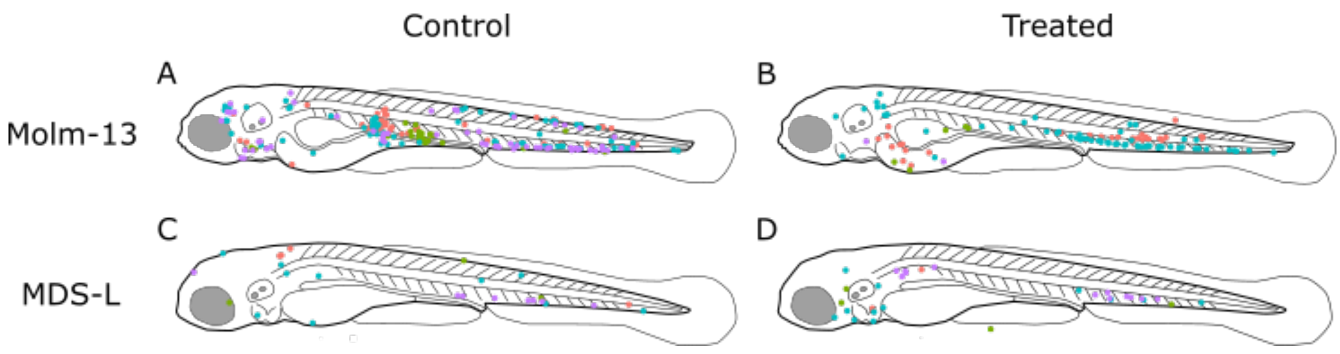

Fig. S4. Inter-larval variation in distribution of transplanted leukaemia cells. Four nL of a $10 \cdot 10^6$ cells$\cdot$ml^{-1} cell suspension containing CellTracker™ Deep Red stained cells of either the Molm-13 or MDS-L cell line were injected into the posterior cardinal vein of zebrafish larvae at two days post fertilization. For Molm-13 injected zebrafish larvae, treatment consisted of a single dose of 4 nL 1mM daunorubicin administered into the PCV the same day as cell transplantation. Zebrafish larvae transplanted with MDS-L received daily injections of 4 nL 1mM azacitidine. To illustrate variations between replicas, the cell locations of four larvae in the same group are shown as dots of different colour. A and B show Molm-13 injected larvae at three days post injection without (A) or with (B) DNR treatment and C and D show larvae injected with MDS-L at three days post injection without (C) or with (D) Aza treatment.

Biology Open • Supplementary information

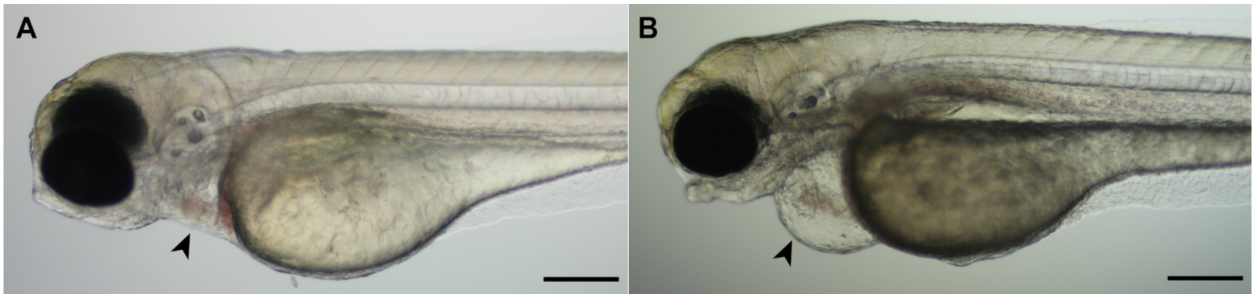

Fig. S5. Illustration of pericardial oedema following azacitidine treatment. Zebrafish larvae were imaged at four days post fertilization after intravenous injections with 4 nL of either PBS (A) or 1 mM azacitidine solution(B) at two- and three days post fertilization. The arrows in A and B indicate the heart and pericardial oedema respectively. The scale bars indicate 200 µm.
